# Gestational constipation and the gut microbiota: mechanisms, interventions, and implications for maternal and offspring health

**DOI:** 10.3389/fnut.2026.1819825

**Published:** 2026-05-08

**Authors:** Linyan Cai, Jinxiao Xie, Ting Luo, Yingying Mao, Xiao Chen, Jiajia Yan, Luyi Yan, Enfu Tao

**Affiliations:** 1 Department of Obstetrics, Wenling Maternal and Child Health Care Hospital, Wenling, Zhejiang, China; 2 Department of Delivery Room, Wenling Maternal and Child Health Care Hospital, Wenling, Zhejiang, China; 3 Department of Neonatology and NICU, Wenling Maternal and Child Health Care Hospital, Wenling, Zhejiang, China

**Keywords:** dietary fiber, gestational constipation, gut microbiota, perinatal health, probiotics, short-chain fatty acids

## Abstract

Gestational constipation affects up to 40% of pregnant women, yet remains underappreciated as a clinical entity with implications beyond maternal discomfort. This review synthesizes current evidence on the intricate relationships between pregnancy-associated constipation, gut microbiota dynamics, and perinatal health outcomes. Pregnancy induces profound remodeling of the maternal gut ecosystem–characterized by reduced alpha diversity, expansion of Actinomycetota and Pseudomonadota, and increased Bifidobacterium–driven by hormonal, immunological, and dietary factors. When perturbed, these physiological shifts transition to dysbiosis, impairing colonic function through deficient short-chain fatty acid production, disrupted serotonergic signaling, and compromised intestinal barrier integrity. Dietary fiber intervention, critically dependent on physicochemical properties (solubility, fermentability), restores microbial equilibrium, alleviates constipation, and exerts systemic anti-inflammatory effects. Probiotic and synbiotic strategies offer strain-specific benefits extending to gestational diabetes mitigation and improved mental wellbeing. Translational evidence from swine models demonstrates that microbiota-targeted interventions during late gestation enhance reproductive performance, colostrum quality, and offspring health—effects mediated through vertical transmission and metabolic programming. Epidemiological studies link maternal constipation to increased risks of offspring atopic dermatitis (1.26-fold) and allergic rhinitis (1.20-fold), while low fiber intake associates with neurodevelopmental delays. Moving beyond one-size-fits-all approaches, future prenatal care must integrate personalized, microbiota-targeted strategies based on predictive microbial biomarkers, optimizing both maternal wellbeing and long-term child health across two generations.

## 1 Introduction

Pregnancy represents a profound physiological state characterized by a series of orchestrated adaptations designed to support fetal growth and development. Beyond the visible anatomical and hormonal changes, this period is marked by significant, albeit less apparent, alterations in the maternal internal ecosystem, particularly within the gastrointestinal tract (1, 2). Two interlinked phenomena commonly emerge during this critical time: a high prevalence of functional gastrointestinal disturbances, notably constipation, and a dynamic remodeling of the gut microbiota (1, 3, 4).

Epidemiological data indicate that constipation affects up to 40% of pregnant women—approximately twice the rate observed in non-pregnant controls—and often persists into the early postpartum period, exceeding 50% prevalence shortly after delivery (4). Concurrently, pregnancy induces substantial shifts in the composition and function of the gut microbiome, a complex community of microorganisms that exists in a symbiotic and interdependent relationship with the host (3, 5, 6). These microbial populations are not passive bystanders but active participants in modulating host metabolism, immune function, and intestinal homeostasis (6, 7).

The convergence of these two elements—widespread constipation and a uniquely evolving gut microbiota—suggests a potential pathogenic link that extends beyond localized bowel dysfunction. The gut microbiota during pregnancy undergoes specific alterations that are thought to promote maternal metabolic adaptations beneficial for fetal development (1, 3). However, disturbances in this delicate microbial balance, or dysbiosis, can disrupt intestinal function and have been implicated in various pregnancy-related complications, including gestational diabetes mellitus (GDM), pre-eclampsia, and preterm birth (PTB) (1–3, 7, 8). Constipation may thus be a clinical manifestation of underlying dysbiosis or a contributor to it, creating a cycle that could impact both maternal and fetal health (9–11). The maternal gastrointestinal environment is increasingly recognized as a fundamental determinant of offspring health, influencing processes ranging from immune system maturation to neurodevelopment (12–14). The maternal-fetal gut microbiota axis emerges as a critical pathway through which prenatal exposures, including dietary patterns and maternal gut health, can program long-term health outcomes in the child (14–17).

This bidirectional relationship underscores the importance of viewing pregnancy not in isolation but as an integrated continuum involving the mother, her microbiome, and the developing fetus. Physiological changes during pregnancy, including differences in hormonal levels, can increase susceptibility to various conditions, such as PTB, preeclampsia, and low birth weight, a concept well-documented in the context of the oral microbiome and its link to adverse pregnancy outcomes (18). Similarly, perturbations in the gut ecosystem may have far-reaching consequences. The process of microbial transmission, colonization, and succession begins even before birth, with the perinatal period being crucial for establishing the infant's microbiome, which in turn influences development during the first critical years of life (12, 17, 19). Therefore, understanding the interplay between maternal constipation and the gut microbiota is essential, not only for alleviating a common maternal complaint but also for optimizing the prenatal environment to support healthier pregnancies and long-term child development (14, 15, 20). This review aims to synthesize current knowledge on this triad, exploring the physiological shifts in the gut microbiota during gestation, the pathophysiology of constipation from a microbiota-centric perspective, and the potential of dietary and microbial interventions to break this cycle for the benefit of both mother and offspring.

## 2 The dynamic landscape of the gut microbiota during pregnancy: physiological shifts and implications

Pregnancy represents a profound physiological state that orchestrates extensive adaptations across multiple maternal systems to support fetal development. Among these adaptations, significant and orchestrated changes occur within the maternal gut microbiota, a complex ecosystem whose composition and function are intricately linked to host metabolism, immunity, and overall health. These gestational microbial shifts are not merely incidental but are increasingly recognized as integral components of a healthy pregnancy, with deviations from expected patterns associated with adverse outcomes. Understanding this dynamic landscape is fundamental to appreciating the context in which gastrointestinal complaints such as constipation arise and to identifying potential avenues for intervention.

### 2.1 Trajectory of gut microbiota composition across gestation

The trajectory of gut microbiota composition across gestation follows a recognizable pattern characterized by distinct taxonomic and ecological alterations. A consistent observation across studies is a reduction in microbial alpha diversity (within-sample diversity) as pregnancy progresses (1, 3). Concurrently, beta diversity (between-sample dissimilarity) increases, indicating a divergence in microbial community structure among pregnant individuals (1). At the taxonomic level, pregnancy is typically associated with an expansion of the phyla Pseudomonadota (formerly Proteobacteria) and Actinomycetota (formerly Actinobacteria), alongside an increase in the relative abundance of specific genera such as *Bifidobacterium* (1, 3). These changes are thought to reflect the evolving metabolic and immunological milieu of the host. Alterations in the abundance of Actinobacteria, Lachnospiraceae, *Akkermansia, Bifidobacterium, Streptococcus*, and *Anaerotuncus* have been specifically linked to the stage of gestation (6). Collectively, these shifts create a microbial profile that enhances energy harvest and promotes metabolic adaptations necessary for sustaining pregnancy, though dysregulation may predispose to insulin resistance.

### 2.2 Drivers of microbial transitions

These microbial transitions are driven by a confluence of hormonal, immunological, metabolic, dietary, and environmental factors inherent to pregnancy (1, 2). Hormonal fluctuations, particularly rising levels of progesterone and estrogen, can directly and indirectly influence gut motility, secretion, and permeability, thereby contributing to constipation development while also altering the habitat for resident microbes. The maternal immune system undergoes a carefully calibrated modulation to tolerate the semi-allogeneic fetus, a state that also impacts the local intestinal immune environment and its interaction with the microbiota. Maternal diet emerges as a powerful and modifiable determinant of microbial ecology during this period. Higher intake and plasma concentrations of carotenoids, often markers of a diet rich in fruits and vegetables, are positively correlated with greater fecal microbial alpha diversity in pregnant women (5). Specific dietary components are associated with the abundance of certain bacterial taxa; intake of total fat and unsaturated fatty acids correlates with *Ruminococcus* and *Paraprevotella*, while protein intake is linked to *Collinsella* and *Anaerostipes* (6). Maternal metabolic status significantly shapes the gut microbiota. Obesity during pregnancy is associated with an increased abundance of Lachnospiraceae, *Bilophila, Dialister*, and *Roseburia*, and levels of *Bilophila* show positive correlations with maternal body mass index, fat mass, and serum triglycerides and insulin (6). This underscores a tight, bidirectional relationship between host metabolism and microbial community structure.

### 2.3 From physiological shifts to dysbiosis and obstetric complications

When perturbed, physiological microbial shifts can transition into dysbiosis linked to several obstetric complications. Alterations in the gut microbiota have been implicated in the pathogenesis of GDM, hypertensive disorders like pre-eclampsia, and PTB (1, 2, 7). Although patterns can be inconsistent, the association suggests that the gut microbiome may influence systemic inflammation, insulin signaling, and placental function. For instance, dysbiosis may contribute to metabolic endotoxemia and low-grade inflammation, which are key features in GDM development (7). Similarly, the oral microbiome undergoes changes during pregnancy, with increased total microbial load and shifts that can contribute to gingivitis and periodontitis, conditions themselves linked to an elevated risk of adverse outcomes like PTB (18). This highlights that the implications of microbial changes extend beyond the gut, encompassing other body sites in a systemic manner. Maternal immune activation driven by infections or inflammatory stimuli, which can be influenced by microbial dysbiosis, has been robustly linked to disruptions in fetal neurodevelopment and an increased risk for neurodevelopmental disorders in offspring (8). Thus, the state of the maternal microbiota holds consequences that bridge maternal and fetal health.

### 2.4 Impact on infant microbiome colonization and long-term health

The ramifications of the maternal gut microbial environment extend into the perinatal period and fundamentally influence the initial colonization and succession of the infant's microbiome. The process of microbial transmission from mother to offspring begins before birth and is shaped by the maternal microbiota at various body sites (17). The composition of the maternal gut microbiota during pregnancy can therefore affect the pioneer microbial communities that colonize the infant, setting the trajectory for the development of the infant's immune and metabolic systems (12, 15). As discussed by Smith, this process can be conceptualized as a form of maternal microbial “handshaking” or “calibration” of the fetal microbiota, wherein the maternal gut ecosystem actively programs offspring health (21). Early-life gut microbiota, established during the critical first 1,000 days from conception to two years of age (22), is critical for long-term health, and imbalances during this formative period are associated with an increased risk of chronic conditions such as asthma, allergic diseases, obesity, and neurodevelopmental disorders later in life (12). This establishes the maternal gut microbiota as a key environmental factor in the developmental origins of health and disease, where its modulation during pregnancy could have intergenerational health benefits. Observations from non-human primate models support the existence of conserved, health-associated microbial taxa across host species, such as butyrate-producing genera like *Faecalibacterium* and *Roseburia*, suggesting fundamental roles for these bacteria in host physiology that may be relevant during life stages including pregnancy (19).

**Figure 1:**
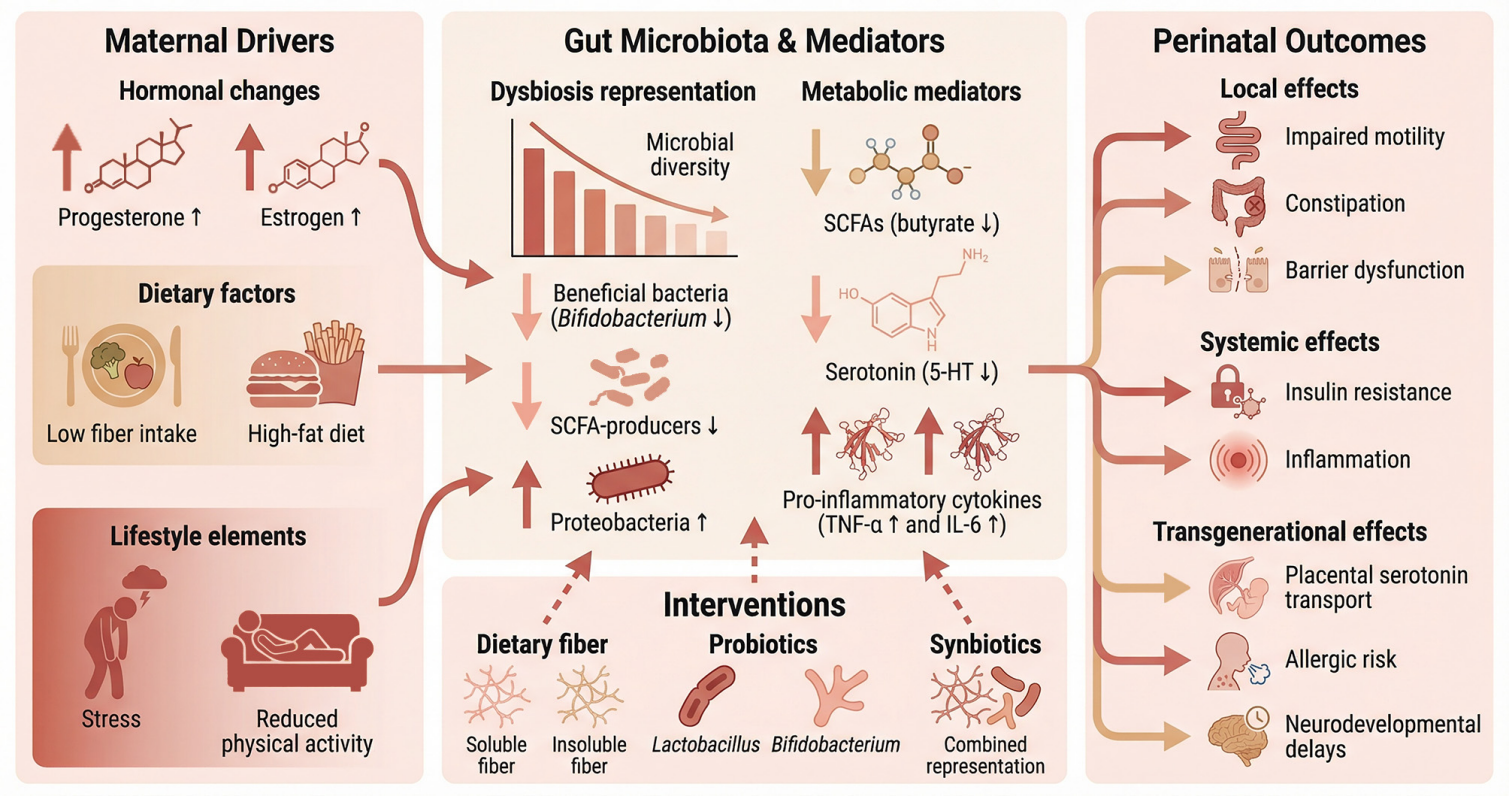
Gut-Brain-Immune Axis in Gestational Constipation. This conceptual framework illustrates the bidirectional relationships between maternal factors, gut microbiota dynamics, and perinatal outcomes. **(Left panel)** (drivers): hormonal changes (progesterone ↑, estrogen ↑), dietary factors (low fiber intake, high-fat diet), and lifestyle elements (stress, reduced physical activity) converge to shape the gestational gut ecosystem. **(Central panel)** (microbiota & mediators): dysbiosis is characterized by reduced *Bifidobacterium* and SCFA-producing genera, increased Proteobacteria, and diminished microbial diversity. Key metabolic mediators include short-chain fatty acids (SCFAs, particularly butyrate ↓), serotonin (5-HT ↓), and pro-inflammatory cytokines (TNF-α ↑, IL-6 ↑). **(Right panel)** (outcomes): these microbial and metabolic alterations drive local effects (impaired motility, constipation, compromised barrier function), systemic effects (insulin resistance, low-grade inflammation), and transgenerational effects via the maternal-fetal axis. Altered placental serotonin transport and immune programming influence fetal neurodevelopment and increase offspring risk for allergic diseases and neurodevelopmental delays. Bottom panel (interventions): targeted interventions—dietary fiber (soluble/insoluble), probiotics (*Lactobacillus, Bifidobacterium*), and synbiotics—restore microbial equilibrium, offering therapeutic potential at multiple nodes of this axis.

In summary, pregnancy induces a dynamic and purposeful recalibration of the maternal gut microbiota. This shift is characterized by reduced alpha diversity, increased beta diversity, and taxonomic changes that support the metabolic demands of gestation. Driven by hormonal, immune, and dietary factors, this altered microbial landscape is a double-edged sword: while adaptive in normal pregnancy, its dysregulation is intimately connected to a spectrum of maternal complications and can influence the foundational health of the offspring. This establishes the gestational gut microbiota not as a passive bystander but as an active participant in reproductive physiology, whose equilibrium is essential for optimal pregnancy outcomes and long-term child health. The mechanistic pathways through which microbiota dysregulation drives constipation and broader perinatal outcomes are illustrated in Figure 1.

## 3 Pathophysiology of constipation in pregnancy: a gut microbiota-centric perspective

As established in Section 2, the gut microbiota undergoes physiological and adaptive changes during normal pregnancy. The following section focuses on pathological deviations from this baseline—specifically, how additional perturbations driven by dietary, lifestyle, or other factors lead to dysbiosis and contribute to the pathophysiology of constipation.

The adaptive yet precarious shifts in the gestational gut microbiota set the stage for understanding how its dysregulation can manifest in common gastrointestinal complaints, most notably constipation (10, 11). Clinically, stool consistency is commonly assessed using the Bristol Stool Scale, a validated instrument that classifies stools into seven types, with types 1 and 2 indicating constipation (23). While traditional explanations for pregnancy-related constipation focus on mechanical pressure from the gravid uterus and the smooth muscle-relaxing effects of elevated progesterone, a microbiota-centric perspective reveals a more intricate pathophysiology. This view posits that the pregnancy-altered microbial ecosystem, when further perturbed by dietary and lifestyle factors, can directly impair colonic function through multiple interdependent mechanisms (24–26), transforming a common physiological adjustment into a symptomatic disorder. The etiology of functional constipation, prevalent in pregnancy, is increasingly linked to an abnormal gut microbiota, with the relationship between microbial populations and gut transit being fundamentally bidirectional (9).

### 3.1 The dysbiotic signature of constipation

Characterizing the dysbiotic state associated with constipation reveals consistent patterns. In non-pregnant populations with functional constipation or constipation-predominant irritable bowel syndrome (IBS-C), alterations often include an increased Firmicutes/Bacteroidetes ratio and changes in specific bacterial genera (11). A key functional consequence is an altered production of short-chain fatty acids (SCFAs)—primarily acetate, propionate, and butyrate—which are crucial metabolites derived from bacterial fermentation of dietary fiber (27, 28). SCFAs serve as the primary energy source for colonocytes, regulate colonic motility and secretion, strengthen the intestinal barrier, and exert potent anti-inflammatory effects (27). In constipation, reduced microbial fermentation capacity, often linked to low-fiber diets, can lead to diminished SCFAs levels, which may contribute to slowed colonic transit, reduced stool bulk, and impaired mucosal health (9, 28). It is important to note that the evidence described in this subsection is primarily derived from non-pregnant populations with functional constipation or IBS-C, as well as from animal models; direct data in pregnant women remain limited.

### 3.2 Dietary context and inflammation

The dietary context is paramount, as a pro-inflammatory diet, characterized by a high Dietary Inflammatory Index (DII) score, is associated with a gut microbiota profile that favors constipation (19, 26). Such a diet correlates with lower abundances of bacteria like *Hungatella* spp. and *Bacteroides fragilis* (the latter positively correlated with anti-inflammatory IL-10) and higher abundances of other *Bacteroides* species (19). Furthermore, dietary patterns with high inflammatory potential have been shown to exacerbate systemic low-grade inflammation, which may further compromise intestinal barrier function and motility (26, 29). Of note, the evidence cited in this subsection is primarily derived from non-pregnant populations and general adult cohorts; direct evidence in pregnant women remains limited.

### 3.3 Microbiota-neurotransmitter interactions

The gut microbiota intricately influences neurotransmitters essential for gut-brain axis communication and local enteric nervous system function. Microbial communities are involved in the synthesis and metabolism of serotonin (5-hydroxytryptamine, 5-HT), a key neurotransmitter that regulates gut motility and secretion (9, 30). Dysbiosis can disrupt serotonergic signaling, which may compromise the neural coordination required for normal defecation (9). This intersection of microbial metabolites and host neuroendocrine pathways illustrates how microbiota dysregulation can affect gastrointestinal function. The pathophysiology thus involves interconnected events linked to a dysbiotic microbiota. Reduced SCFA production may decrease colonic motility and disrupt water absorption, while altered neurotransmitter signaling could further impair motor function. Concurrently, a weakened intestinal barrier, partly due to low butyrate levels, may permit low-grade inflammation, which can dysregulate gut function (27). This can create a self-perpetuating cycle: constipation leads to prolonged colonic transit time, altering the physicochemical environment within the gut, which in turn selects for a microbiota that may exacerbate the condition. Stool retention reduces the availability of fermentable substrate for beneficial bacteria, potentially favoring the growth of microbes whose metabolites, like methane, can slow intestinal transit (9). It should be noted that the mechanistic insights described in this subsection are primarily derived from non-pregnant populations and animal models; whether these pathways operate identically during pregnancy requires further investigation.

### 3.4 Intervention evidence

Intervention studies support the role of the microbiota in this pathophysiology. For instance, supplementation with psyllium husk not only alleviated symptoms but also significantly altered gut microbiota composition and enriched microbial metabolic pathways in chronically constipated women of reproductive age (31). Psyllium is a soluble bulk-forming fiber that operates through a distinct physiological mechanism without stimulating intestinal smooth muscle contraction. Importantly, unlike stimulant laxatives (e.g., bisacodyl, senna), bulk-forming fibers such as psyllium are not associated with the adverse offspring outcomes discussed later in this review (32). This demonstrates that a substrate which directly feeds the microbiota can correct both the microbial imbalance and its functional consequences. Probiotics are thought to ease constipation by modulating this ecosystem, strengthening the intestinal barrier, and modulating immune and neural responses (10).

Therefore, drawing on mechanistic insights from non-pregnant populations and animal models, we propose that the pathophysiology of constipation in pregnancy can be reframed as a condition where the inherent physiological shifts in the gut microbiota intersect with dietary predispositions. Direct evidence in pregnant populations is needed to confirm this framework. This confluence can tip the balance from a compensated state to a dysbiotic one characterized by deficient SCFA production, disrupted gut-brain signaling, and compromised barrier function. This microbiota-centric model provides a cohesive framework linking dietary intake, microbial metabolism, and host gastrointestinal physiology, offering novel targets for therapeutic intervention aimed at restoring microbial equilibrium to relieve symptoms.

## 4 Dietary fiber intervention: modulating gut microbiota to alleviate constipation and improve pregnancy outcomes

Dietary fiber intervention emerges as a core strategy to restore the microbial equilibrium disrupted in pregnancy-associated constipation. The efficacy of this intervention is critically dependent on the specific physicochemical properties of the fiber source—such as solubility, viscosity, and fermentability—which dictate its interaction with the gut microbiota (24, 25).

### 4.1 The fiber intake gap

A fundamental challenge is the widespread inadequacy of fiber intake among pregnant individuals. Studies show that a majority fail to meet recommended daily intake levels, with one analysis reporting that 84.7% of participants in their third trimester did not meet dietary fiber guidelines (33). This deficiency is significant because general fiber intake during pregnancy shows a positive association with gut microbial alpha diversity (33, 34).

### 4.2 Mechanisms of action: fermentation and SCFAs

The physiological effects of dietary fiber are mediated through its fermentation by the colonic microbiota. Soluble fibers, such as inulin, isomalto-oligosaccharides (IMO), konjac flour, and certain resistant starches, are readily fermented by gut bacteria (35–37). This fermentation process generates SCFAs, principally acetate, propionate, and butyrate (28). In pregnant sow models, supplementation with fermentable fibers like potato resistant starch (PRS) and konjac flour (KON) significantly increased serum levels of gut motility regulators like 5-HT and motilin, concurrently elevating fecal butyrate concentrations and enriching SCFA-producing genera such as *Bacteroides, Parabacteroides*, and *Turicibacter* (36, 38). A positive correlation was observed between defecation frequency, serum 5-HT, and the abundance of *Turicibacter* and butyrate, underscoring a direct mechanistic pathway (38). *In vitro* fermentation studies demonstrate that different soluble fibers yield distinct SCFA profiles and microbial community shifts over time (37, 39). The physicochemical properties and physiological effects of common dietary fibers in pregnancy are summarized in Table 1.

**Table 1:** Physicochemical properties and physiological effects of common dietary fibers in pregnancy.

Fiber type	Source examples	Solubility	Fermentability	Key microbiota shifts	SCFA profile	Effect on constipation	Systemic effects	Key references
Resistant starch	Potato, corn, High-amylose maize	Soluble	High	*Bacteroides* ↑, *Parabacteroides* ↑, *Turicibacter* ↑	Butyrate ↑↑	Significant improvement	Serum 5-HT ↑, motilin ↑, IL-10 ↑	(36, 38, 43)
Inulin	Chicory root, Jerusalem artichoke	Soluble	High	*Bifidobacterium* ↑, *Lactobacillus* ↑, *Faecalibacterium* ↑	Acetate ↑, Butyrate ↑	Improvement	Reduced inflammation, Improved insulin sensitivity	(35, 36, 47)
Konjac flour	Konjac root (*Amorphophallus konjac*)	Soluble (viscous)	High	*Parabacteroides*↑, *Prevotella* ↑	Propionate ↑, Butyrate ↑	Significant improvement	Serum 5-HT ↑↑, motilin ↑, TNF-α ↓	(36, 38)
Isomalto-oligosaccharides (IMO)	Starch hydrolysate	Soluble	High	*Bifidobacterium*↑, *Lactobacillus* ↑	Acetate ↑, Propionate ↑	Improvement	Improved reproductive performance	(35, 36)
Sugar beet Pulp	Sugar beet processing byproduct	Mixed (soluble + insoluble)	Moderate-high	Microbial diversity ↑, *Roseburia* ↑	Total SCFAs ↑	Improvement	IL-10 ↑, TNF-α ↓, Cholesterol ↓	(38, 42)
Psyllium husk	*Plantago ovata* seeds	Soluble (mucilaginous)	Low-moderate	*Bacteroides* ↑, *Prevotella* ↑	Acetate ↑	Improvement (stool consistency)	LDL cholesterol ↓	(31)
Wheat bran	Wheat milling byproduct	Insoluble	Low-moderate	Cellulolytic bacteria ↑	Minimal impact	Mild improvement	Minimal systemic effects	(38, 40, 42)
Lignocellulose	Wood, plant fibers	Insoluble	Very low	Minimal diversity change	Minimal SCFAs production	Bulk-forming only	No significant anti-inflammatory effect	(38, 40)
Sugarcane bagasse	Sugarcane processing residue	Insoluble	Low	Diversity ↓, *Desulfovibrio* ↑	Branched-chain SCFAs ↓	May worsen constipation (at high doses)	Negative metabolic effects at high inclusion	(41)
Bamboo fiber	Bamboo shoots/stems	Mixed	Moderate	*Lachnospira* ↑, *Roseburia* ↑	Butyrate ↑	Improvement	Serum lipids ↓, Antioxidant effects ↑	(43, 45, 46)

↑ indicates increase; ↓ indicates decrease; ↑↑ indicates strong increase. SCFAs, short-chain fatty acids; 5-HT, serotonin; IL-10, interleukin-10; TNF-α, tumor necrosis factor-alpha; LDL, low-density lipoprotein.

### 4.3 The double-edged sword: fiber source and dose matter

The physicochemical properties of fiber also determine its physical role in the gut. Bulky, insoluble fibers like lignocellulose can increase fecal bulk and water-holding capacity (40). However, an inappropriate source or dose can be counterproductive. High inclusion rates of sugarcane bagasse (10–15%) in sow diets significantly increased constipation rates, disturbed gut microbial diversity, and reduced fecal concentrations of beneficial branched-chain SCFAs, highlighting that the source and dose are essential (41).

### 4.4 Systemic anti-inflammatory effects

Beyond local gastrointestinal effects, the modulation of the gut microbiota by dietary fiber has systemic anti-inflammatory consequences relevant to pregnancy. SCFAs, particularly butyrate, are known to enhance gut barrier integrity and exert anti-inflammatory effects (28). In sows, fibers like sugar beet pulp and resistant starch have been shown to increase anti-inflammatory cytokine IL-10 and decrease pro-inflammatory TNF-α in serum (38, 42). Human data show that higher dietary fiber intake is inversely associated with systemic immune-inflammatory biomarkers, including neutrophil-to-lymphocyte ratio (NLR) and high-sensitivity C-reactive protein (hs-CRP) (29). A pro-inflammatory diet is associated with a gut microbiota profile linked to constipation, while an anti-inflammatory diet correlates with better bowel function (26). This establishes a clear diet-microbiota-inflammation axis wherein fiber intake can mitigate the low-grade inflammatory state often observed in pregnancy.

### 4.5 Beyond constipation: enhancing reproductive and offspring outcomes

**Figure 2:**
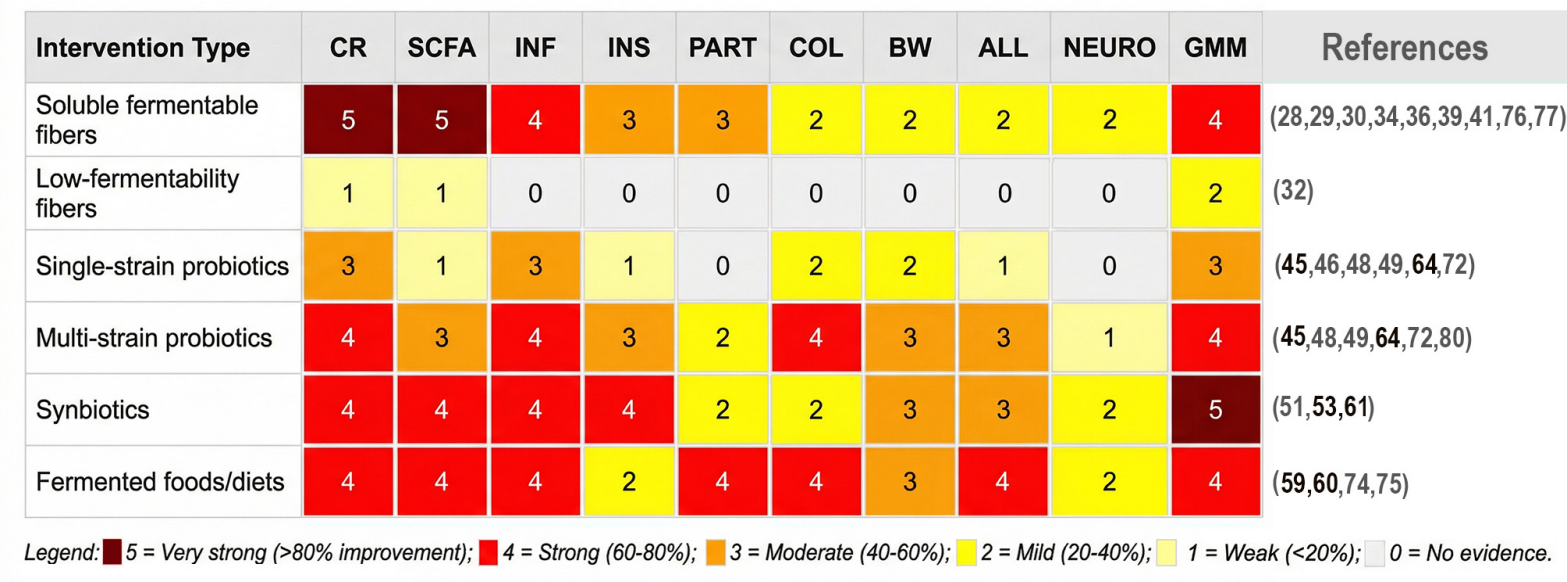
Multi-dimensional effects of microbiota-targeted interventions in pregnancy. This heat map synthesizes evidence from human studies and translational animal models (primarily swine) on the efficacy of dietary fiber, probiotic, synbiotic, and fermented diet interventions across maternal and offspring outcomes. Color intensity represents magnitude of effect, with darker shading indicating greater improvement (5 = dark red, 4 = red, 3 = orange, 2 = yellow, 1 = light yellow, 0 = light gray). Key observations include: (i) Soluble fermentable fibers (resistant starch, inulin, konjac flour) show strongest effects on constipation relief and SCFA production; (ii) Multi-strain probiotics and synbiotics demonstrate broader systemic effects, including inflammation reduction and metabolic benefits; (iii) Fermented diets exert the most pronounced transgenerational effects, particularly on offspring immune development and gut microbiota maturation; (iv) Low-fermentability fibers (wheat bran, lignocellulose) have minimal impact on most outcomes, limited to mild bulk-forming effects. CR, constipation relief; SCFA, short-chain fatty acid; INF, inflammation reduction (IL-10↑/TNF-α↓); INS, insulin sensitivity; PART, parturition duration; COL, colostrum quality; BW, birth weight/growth; ALL, allergy risk reduction; NEURO, neurodevelopment; GMM, gut microbiota maturation.

The benefits of strategic fiber intervention extend beyond alleviating maternal constipation to directly enhancing reproductive and developmental outcomes. In swine models, fermentable fiber supplementation has improved key performance metrics, including increased litter size, piglet birth weight, and reduced rates of stillbirth and intrauterine growth restriction (IUGR) (36, 38, 43, 44). The proposed mechanisms are multifaceted. One pathway involves the serotonergic system: fiber fermentation increases colonic SCFAs, which may upregulate local 5-HT synthesis; this maternal serotonin can be transported to the placenta, where it promotes placental development and function (43). Another pathway involves bile acid metabolism, where specific soluble fibers can modulate bacterial populations leading to altered bile acid profiles and improved reproductive performance (44). Furthermore, fiber-induced improvements in maternal metabolism, evidenced by lowered serum cholesterol and triglycerides, contribute to a healthier gestational milieu (42, 45). Emerging human evidence indicates that low maternal dietary fiber intake during pregnancy is associated with a higher risk of neurodevelopmental delays in offspring (20). This underscores the potential long-term impact of maternal fiber nutrition. Figure 2 provides a comprehensive summary of the multi-dimensional effects of microbiota-targeted interventions on maternal, perinatal, and offspring outcomes, illustrating the superior efficacy of combined approaches and the critical importance of intervention timing. Future research must focus on elucidating strain-specific mechanisms and optimizing formulations for personalized microbiota-targeted care.

### 4.6 Optimizing fiber selection

The selection of the optimal fiber type and dose is a critical consideration. Research indicates synergistic effects when combining soluble and insoluble fibers, as their distinct properties can complement each other in modulating gut function and metabolism (46). The goal is to foster a microbial ecosystem conducive to gastrointestinal motility and systemic health. This includes promoting bacteria associated with SCFA production and reducing the abundance of pro-inflammatory taxa (38, 41, 47). Notably, dietary patterns rich in diverse fibers are also associated with a lower burden of antimicrobial resistance genes (ARGs) in the gut microbiome (48). Ultimately, dietary fiber intervention represents a powerful, microbiota-centric approach to managing constipation in pregnancy. By strategically selecting fibers based on their physicochemical properties, it is possible to correct dysbiosis, amplify SCFA production, dampen inflammation, and activate host physiological pathways that collectively relieve constipation and support maternal and offspring health. The multi-dimensional effects of these interventions on maternal and offspring outcomes are summarized in Figure 2.

## 5 Probiotic and synbiotic strategies: targeting the gut microbiota for gastrointestinal and systemic benefits in pregnancy

Building upon the role of dietary fibers, the direct administration of specific live microorganisms, or probiotics, presents a targeted approach to correct dysbiosis and address constipation in pregnancy.

### 5.1 Clinical evidence for probiotics in constipation

Clinical evidence demonstrates that probiotic combinations can effectively alleviate pregnancy-associated constipation, as shown by improvements in defecation frequency, time, and stool consistency (49). This symptomatic relief is underpinned by a restoration of microbial diversity and a shift toward a more favorable community structure, characterized by increased relative abundances of beneficial genera such as *Faecalibacterium, Bifidobacterium*, and *Phascolarctobacterium* (49). The efficacy of specific strains is further supported by *in vitro* models showing their ability to increase the abundance of beneficial bacteria in the fecal microbiota of constipated pregnant women (50).

### 5.2 Mechanisms of action

The mechanisms through which probiotics confer benefits extend beyond compositional changes to include functional modulation of the gut environment and host responses. Probiotics can inhibit the colonization of pathogens, enhance the intestinal mucosal barrier, and modulate the host immune system (51). This immunomodulatory capacity is relevant in pregnancy. Probiotics can interact with intestinal immune cells to promote a balanced immune response, often characterized by a shift toward anti-inflammatory pathways (52, 53). By decreasing gut dysbiosis and intestinal permeability, probiotic therapy may minimize the activation of systemic inflammatory pathways (53). This anti-inflammatory action aligns with observations in late-gestating sows where probiotic supplementation alleviated both constipation and systemic inflammation (54).

### 5.3 Impact on obstetric complications

The physiological shifts in the gut microbiota during pregnancy create a unique context for probiotic intervention (1). Beyond alleviating gastrointestinal distress, modulating this ecosystem holds promise for mitigating obstetric complications. A prominent focus has been on GDM, where gut microbiota dysbiosis is implicated. Systematic reviews indicate that probiotics may help prevent the occurrence of GDM and, in those diagnosed, improve glycemic control and markers of inflammation (55–57). These supplements have also been associated with improved neonatal outcomes, such as reduced risks of fetal hyperbilirubinemia and macrosomia (57). However, results are not uniform, with some trials reporting no significant reduction in GDM incidence, underscoring the influence of factors like probiotic strain and population (58).

### 5.4 Maternal mental health

The impact of probiotics in pregnancy may also influence maternal mental wellbeing through the gut-brain axis. A recent meta-analysis by Halemani et al. including 12 randomized controlled trials found that probiotic supplementation significantly reduced anxiety symptoms in pregnant and lactating women, although the effect on depressive symptoms was less consistent, with subgroup analyses suggesting potential benefits only in certain populations (59).

Several independent randomized controlled trials have further supported these findings. Hulkkonen et al. demonstrated that supplementation with a multi-strain probiotic (*Lactobacillus rhamnosus* HN001 and *Bifidobacterium animalis* subsp. *Lactis* 420) from early pregnancy to 6 months postpartum significantly reduced anxiety scores at 6 months postpartum compared to placebo in overweight and obese women (60). Similarly, a double-blind pilot trial by Browne et al. reported that a 9-week intervention with *Lactobacillus rhamnosus* HN001 during the third trimester was associated with lower anxiety scores at 30 weeks gestation and 6 weeks postpartum (61). A systematic review by Desai et al. corroborated these findings, concluding that probiotic supplementation, particularly with strains of *Lactobacillus* and *Bifidobacterium*, may offer a safe and accessible strategy to improve perinatal mental health, though they emphasized the need for larger, well-powered trials to confirm optimal strains and dosages (62).

Collectively, these findings suggest that modulation of the gut-brain axis via probiotic supplementation holds promise as a non-pharmacological intervention to support maternal mental health during pregnancy and the postpartum period.

### 5.5 Translational evidence from animal models

Insights from animal models provide translational evidence for the multi-system benefits of probiotics. Supplementation with specific strains in sow diets has been shown to improve maternal performance and offspring health (63, 64). These benefits are correlated with improved gut microbiota profiles, increased production of SCFAs, and favorable modulation of systemic immune markers (54, 64). Furthermore, specific probiotic strains have demonstrated the ability to enhance intestinal barrier function and nutrient utilization (65). These findings mirror potential cross-generational impacts in humans, where maternal probiotic intake may influence infant gut microbiota (66).

### 5.6 Synbiotic strategies

Synbiotic strategies, which combine probiotics with prebiotic substrates, aim to create a synergistic effect. This approach can enhance the survival and efficacy of the administered probiotics. In the context of GDM, synbiotic supplements have demonstrated clinical efficacy in improving metabolic parameters and inflammatory markers (57). The prebiotic component independently modulates the gut microbiota, as seen in studies where prebiotic supplementation increased the abundance of beneficial genera and elevated SCFA concentrations (67). When combined with a compatible probiotic, the result is a more robust modulation of the gut ecosystem.

### 5.7 Strain specificity and personalization

Despite promising evidence, the application of probiotics and synbiotics in pregnancy requires consideration of strain-specificity. Their effects are highly dependent on the specific strain or combination used (68, 69). For instance, *Lactobacillus plantarum* 299v has shown efficacy in improving iron status in pregnant women (70). Conversely, the efficacy of a probiotic for a condition like IBS may be predictable based on baseline microbiota features (71). This underscores the future direction toward personalized probiotic interventions. Safety and tolerability in pregnancy are critical, and available data from clinical trials generally report good safety profiles and high acceptability for various probiotic strains (72, 73).

In conclusion, probiotic and synbiotic strategies represent a targeted, microbiota-centric approach to managing constipation and improving broader health outcomes in pregnancy. By directly introducing beneficial microbes and providing substrates for their growth, these interventions can correct dysbiosis, enhance SCFA production, strengthen intestinal barrier function, and exert systemic immunomodulatory effects. The benefits extend from relieving gastrointestinal discomfort to potentially mitigating the risks of GDM, improving perinatal mental health, and positively influencing offspring development. Future research must focus on elucidating strain-specific mechanisms and optimizing formulations for personalized microbiota-targeted care.

## 6 Beyond the gut: impact on offspring health and development

The profound influence of maternal interventions on the gut microbiota extends its significance far beyond alleviating maternal gastrointestinal distress, reaching into the critical realm of offspring health and development.

### 6.1 Epidemiological evidence: allergic diseases

Large-scale epidemiological studies provide compelling evidence linking maternal gastrointestinal health to offspring atopic diseases, a group of disorders rooted in immune dysregulation. A nationwide retrospective cohort study in Taiwan, China demonstrated that maternal constipation was associated with a 1.26-fold increased risk of atopic dermatitis (AD) in offspring (74). This risk exhibited a dose-response relationship, as constipated mothers who required more frequent laxative prescriptions had an even higher adjusted hazard ratio for child AD (74). Similarly, maternal constipation was independently associated with a 1.20-fold increased risk of allergic rhinitis (AR) in children (75). These associations remained significant across various subgroups, accounting for factors like child's sex, birth weight, and mode of delivery, underscoring the robustness of the link. Early-life laxative exposure in infants themselves is also an independent risk factor for subsequent allergic disease development, with prenatal maternal laxative use identified as an independent risk factor, suggesting a continuity of risk from the intrauterine environment into postnatal life (76). Of note, the risks associated with stimulant laxatives (e.g., bisacodyl, senna, and anthraquinone derivatives) are not generalizable to bulk-forming fibers such as psyllium or osmotic laxatives such as lactulose, which operate through distinct mechanisms. The mechanisms underpinning this “gut-skin” (in AD) or “gut-airway” (in AR) axis likely involve the foundational role of the gastrointestinal microbiota in educating and calibrating the developing immune system (77, 78). Perturbations to the maternal gut microbiome, such as those occurring in constipation, may skew immune priming *in utero* or affect the vertical transmission of microbes, setting the stage for impaired immune tolerance and a heightened Th2 response characteristic of allergic diseases (12, 13).

### 6.2 Neurodevelopmental outcomes

Beyond allergy, the impact of maternal gut status touches upon neurodevelopment. A prospective cohort study of over 76,000 mother-infant pairs in Japan revealed that low maternal dietary fiber intake during pregnancy was associated with a significantly higher risk of developmental delay in multiple domains—communication, fine motor, problem-solving, and personal-social skills—in 3-year-old children (20). While fiber deficiency is not synonymous with constipation, both states are frequently characterized by altered gut microbiota composition and reduced production of microbial metabolites like SCFAs. Animal studies support this link, indicating that maternal immune activation (MIA), a state of heightened inflammation often connected to dysbiosis, can disrupt fetal brain development and is implicated in neurodevelopmental disorders (8). The gut microbiota is a key modulator of maternal immune function, and its dysregulation could thus be a conduit through which maternal inflammation influences the fetal brain (8).

### 6.3 Mechanistic insights from animal models

Insights from animal models, particularly swine, offer mechanistic clarity and demonstrate the potential for maternal dietary modulation to positively shape offspring outcomes. These models show that targeting the maternal gut microbiota during gestation can have tangible benefits for the progeny. For instance, feeding sows a fermented diet—rich in live probiotics and metabolites—accelerated the maturation of the neonatal gut microbiota, increased the abundance of beneficial *Lactobacillus*, and reduced susceptibility to colonic inflammation in piglets (79). Direct oral administration of *Lactobacillus plantarum* to sows improved the body weight and health status of their offspring, reducing pre-weaning mortality and diarrhea incidence (63). Supplementation with co-fermented feed containing *Bacillus subtilis* and *Enterococcus faecium* not only reduced sow constipation but also decreased diarrhea in piglets and improved their weight gain (64). Similarly, including fermented mulberry leaves rich in fiber in the gestational diet improved the diversity of sows' gut microbiota and enhanced the weaning litter weight (80). Specific prebiotic interventions, such as dietary inulin, have been shown to regulate the maternal gut microbiota, shorten farrowing duration, reduce the percentage of weak-born piglets, and improve the survival rate and growth performance of suckling neonates (47). These benefits may be mediated through enhanced microbial production of SCFAs, improved intestinal barrier function, and modulation of the maternal immune and metabolic milieu, which in turn influence the intrauterine environment and the composition of breast milk (47, 64, 79). The principle extends to direct postnatal application; for example, the probiotic *Lactobacillus amylovorus* was shown to improve growth and intestinal barrier function in intrauterine growth-restricted piglets, highlighting the continuity of gut microbiota-targeted support from the prenatal to postnatal period (65). Early-life nutritional composition, including prebiotic oligosaccharides, can further shape the infant gut ecosystem, promoting bacterial genera associated with beneficial SCFA production (67).

Collectively, the evidence underscores that maternal constipation and the associated gut dysbiosis are not isolated maternal issues but are significant risk factors for adverse offspring outcomes, particularly allergic diseases and potential neurodevelopmental delays. The consistent findings from human observational studies and supportive interventional data from animal models suggest that the maternal gut microbiome acts as a key interface between maternal health and fetal programming. This establishes a compelling rationale for addressing maternal gastrointestinal health and microbiota composition as a preventive strategy to safeguard not only the mother's wellbeing but also the lifelong health of her child, reinforcing the concept of pregnancy as a critical window for intervention (14, 15, 17).

## 7 Integrative insights from animal models: translating findings from sows to human pregnancy

The compelling links between maternal gut health, microbiota, and offspring development observed in human studies find robust mechanistic validation and causal exploration in controlled animal models. The pregnant sow (*Sus scrofa domesticus*) serves as a particularly valuable and translational model for studying gestational constipation and microbiota dynamics due to striking physiological parallels with human pregnancy. These include a similar length of gestation (~114 days), the development of insulin resistance in late pregnancy to prioritize nutrient partitioning to the fetus, and the critical importance of colostrum quality for neonatal survival and development (81, 82). Furthermore, sows frequently experience late-gestation constipation and related metabolic inflammation, mirroring common clinical complaints in pregnant women, thereby offering a direct path to investigate interventions and underlying mechanisms (38, 54).

### 7.1 Differential effects of dietary fibers

Research utilizing the sow model has systematically elucidated the differential effects of dietary fibers based on their physicochemical properties, moving beyond a simplistic “more fiber is better” paradigm. Studies comparing fibers like PRS, konjac flour (KON, a viscous soluble fiber), and lignocellulose (LIG, an insoluble bulky fiber) have demonstrated that not all fibers are equivalent. Consistent with the findings in humans (as detailed in Section 4.2), studies in the sow model have shown that fermentable fibers such as PRS and KON alleviate constipation by modulating gut motility regulators (e.g., 5-HT) and enriching SCFA-producing genera (38). This underscores the principle that the efficacy of fiber in modulating gut motility is intimately tied to its fermentability and the resulting microbial metabolic output, particularly SCFAs like butyrate (39). Conversely, the use of poorly fermentable fibers like high levels of sugarcane bagasse or inappropriate fiber sources can disturb microbial diversity, deplete beneficial SCFAs like isobutyrate and isovaleric acid, and paradoxically increase constipation rates, highlighting the need for precision in fiber source selection (41). The selection of fiber source and level requires careful consideration, as responses in reproductive performance and nutrient digestibility vary significantly (83).

### 7.2 Multifaceted benefits beyond constipation

Beyond alleviating constipation, fiber supplementation in late gestation consistently demonstrates multifaceted benefits for maternal and offspring health. High-fiber diets improve insulin sensitivity in sows during late pregnancy, a process linked to the modulation of tryptophan metabolism toward serotonin production and associated with favorable microbiota changes (82). These maternal benefits translate into enhanced reproductive outcomes, including shortened farrowing duration, reduced rates of stillbirths and weak-born piglets, and improved colostrum quality (higher immunoglobulins like IgA and IgM, and protein content) (47, 84, 85). The improved colostrum quality and metabolic environment subsequently support superior offspring growth performance and vitality (84, 85). Mechanistic studies further reveal that dietary fiber can promote fetal growth by enhancing placental development and function through a serotonin-signaling pathway, wherein maternal colonic serotonin synthesis and transport to the placenta are upregulated by fiber-induced microbiota changes and SCFA production (43).

### 7.3 Probiotic interventions

Probiotic interventions, both as direct supplements and as components of fermented feeds, offer another potent microbiota-targeting strategy validated in the sow model. Supplementation with specific strains such as *Bacillus subtilis* QST 713 or *Lactobacillus plantarum* CAM6 alleviates perinatal constipation, reduces systemic inflammation (elevating anti-inflammatory IL-10, reducing TNF-α), and reshapes the maternal gut bacteriome and phageome (54, 63, 85). These maternal modifications lead to tangible benefits: shortened parturition, reduced stillbirth rates, improved colostrum immune components, and ultimately, enhanced piglet growth and reduced pre-weaning mortality (63, 64, 85). Fermented diets, which contain live probiotics and their metabolites, exert even broader effects, modulating the maternal milk metabolome (e.g., increasing L-glutamine) and accelerating the maturation of the neonatal gut microbiota, thereby conferring long-term protection against intestinal inflammation in the offspring (79). This demonstrates a clear vertical transmission of benefits from the maternal diet, through microbiota and milk, to the infant's gut health and resilience.

### 7.4 Translational principles for human pregnancy

The integrative insights from these animal studies underscore several key translational principles for human pregnancy. First, the timing of intervention is critical, with late gestation being a pivotal window for modulating microbiota to impact parturition processes and colostrum and milk quality (47, 81, 84). Second, the choice of fiber or probiotic is not arbitrary; specific sources and strains elicit distinct microbial and physiological responses (38, 42, 86). For instance, soluble, fermentable fibers like inulin or certain resistant starches appear particularly effective in promoting SCFA production and gut motility hormones (36, 38, 82). Third, the benefits extend far beyond the relief of gastrointestinal symptoms to encompass systemic metabolic health (insulin sensitivity), reproductive success, and offspring programming (43, 44, 82). Finally, the sow model clearly illustrates that excessive or inappropriate fiber supplementation can be detrimental, emphasizing the need for evidence-based, personalized nutritional guidance rather than blanket recommendations (41, 45).

While the sow model provides unparalleled insights, direct translation to humans requires careful consideration of differences in diet, anatomy, and microbiota composition. The consistent patterns observed—whereby targeted modulation of the maternal gut ecosystem alleviates constipation, reduces inflammation, improves metabolic and reproductive outcomes, and enhances offspring health—provide a strong scientific rationale for designing and testing similar microbiota-centric interventions in human prenatal care. These animal studies effectively deconvolute the complex interactions between diet, microbes, and host physiology, offering a mechanistic roadmap for developing strategies to optimize maternal and child health beginning in the prenatal period. Table 2 summarizes key intervention studies for constipation discussed in this review.

**Table 2:** Summary of key intervention studies for constipation.

Population	Sample size	Intervention	Timing and dosage	Main outcomes	References
Pregnant women with constipation	100 (70 constipated, 30 healthy controls)	Probiotic combination	7 days (1 pack after breakfast and dinner daily)	Significantly improved defecation frequency, stool consistency, and reduced defecation difficulty. Altered gut microbiota composition.	He et al. (49)
Chronically constipated women of reproductive age	54 (placebo: 29, psyllium husk: 25)	Psyllium husk	4 weeks	Alleviated constipation symptoms. Altered gut microbiota composition and enriched metabolic pathways.	Yang et al. (31)
Pregnant sows	30 (10 per group)	Soluble dietary fiber (Inulin and Isomalto-oligosaccharide)	40 days (from day 64 to 104 of gestation); 0.5% in diet	Alleviated constipation, modulated motility-related hormones and short-chain fatty acids, improved fecal microflora, and significantly increased litter size.	Yu et al. (36)
Late-gestation sows	80 (allocated to 4 groups)	Dietary fibers with different properties (e.g., Resistant Starch-PRS, Konjac-KON)	From day 85 of gestation till farrowing; 2% PRS or 2% KON in diet	PRS and KON increased defecation frequency and fecal moisture, improved serum gut motility regulators (5-HT, MTL), reduced inflammation and stillbirths, and optimized gut microbiota. PRS showed the best effect.	Lu et al. (38)
Sows in late gestation and lactation	74 (36 probiotic, 38 control)	Probiotic combination (Probio-M8 and Probio-M9) in drinking water	From 100 days of gestation to 23 days of lactation; 40 g per ton of water	Alleviated constipation, reduced serum pro-inflammatory factors, improved piglet growth. Reshaped gut microbiota and phageome structure.	Ma et al. (54)
Sows from late gestation to lactation	45 (15 per group)	Dietary fiber (Sugar Beet Pulp - SBP, Wheat Bran - WB)	From day 85 of gestation to end of lactation (day 21 post-farrowing); 20% SBP (gestation) and 10% SBP (lactation) or 30% WB (gestation) and 15% WB (lactation)	SBP reduced sow weight loss, lowered blood lipids and inflammatory factors, modulated beneficial microbiota, and increased short-chain fatty acid concentrations more effectively than WB.	Shang et al. (42)
Sows in late gestation and lactation	20 (10 per group)	Probiotic (Lactobacillus plantarum CAM6)	From day 75 of gestation throughout suckling period; basal diet plus 10 mL biological agent containing 10? CFU/mL daily	Did not affect sow reproductive performance but reduced pre-weaning piglet mortality, improved milk composition (increased lactose, non-fatty solids), and increased piglet body weight while reducing diarrhea incidence.	Betancur et al. (63)

CSS, constipation severity scale; PRS, potato resistant starch; KON, konjac; 5-HT, 5-hydroxytryptamine (serotonin); MTL, motilin; SBP, sugar beet pulp; WB, wheat bran; CFU, colony-forming units.

## 8 Conclusions and future directions

The convergence of evidence firmly establishes the maternal gut microbiota as a pivotal interface between physiological adaptations in pregnancy, common gastrointestinal complaints like constipation, and a spectrum of maternal-fetal health outcomes.

### 8.1 Summary of key findings and limitations

Pregnancy orchestrates a profound remodeling of the gut ecosystem, characterized by shifts toward increased *Bifidobacterium, Actinomycetota*, and *Pseudomonadota*, alongside reduced alpha diversity, changes intrinsically linked to metabolic and immune programming (1, 2). These alterations, while physiological, can tilt toward dysbiosis, contributing to the pathophysiology of constipation through mechanisms involving bile acid metabolism, SCFA production, and intestinal motility (9, 10). Concurrently, such dysbiotic states are implicated in the pathogenesis of major pregnancy complications, including GDM and PTB, underscoring the gut microbiome's role as a modulator of systemic gestational health (1, 2, 7). The compelling rationale, therefore, lies in strategically modulating this microbial community to alleviate localized gastrointestinal symptoms and potentially mitigate broader adverse outcomes. Critically, the objective of microbiota-targeted strategies in pregnancy must be conceptualized broadly, aiming not merely for symptomatic relief of constipation but for enduring systemic benefits for both mother and child. The maternal microbiome is a key component of the in utero environment, influencing fetal immune development and priming neonatal microbial colonization (12, 13, 17). Interventions that successfully optimize the maternal gut ecosystem may therefore have a dual impact: improving immediate maternal wellbeing and gastrointestinal function, while potentially reducing the long-term risk of immune-mediated and metabolic diseases in the offspring by promoting healthier developmental trajectories (12, 13). Furthermore, modulation may extend to other body sites; for example, certain probiotics may exert protective effects against PTB by influencing the vaginal microbiota, and early evidence suggests potential benefits for maternal anxiety, highlighting the interconnected nature of the microbiome-gut-brain axis (1, 62, 73). The safety and acceptability of such interventions, including live biotherapeutics, are of utmost importance and initial studies in high-risk pregnancies report favorable profiles, paving the way for larger efficacy trials (73).

However, it is important to acknowledge that direct evidence in pregnant populations remains limited, and most data presented come from animal models or non-pregnant populations. Therefore, considering the low number of studies for constipation in pregnant human populations, and that no grading of the strength of the evidence has been performed in this review, no clinical practice recommendations can be suggested.

### 8.2 The need for personalization

The current paradigm of probiotic and dietary intervention, though promising, is hampered by a “one-size-fits-all” methodology, leading to inconsistent clinical results (68, 69). The efficacy of probiotics is demonstrably strain-specific and outcome-specific, a principle clearly illustrated in gastrointestinal disorders outside pregnancy where certain strains show significant benefits for specific symptoms like abdominal pain, while others do not (69, 87). This heterogeneity of response is likely mirrored in pregnant populations. Emerging research points to the existence of microbial signatures that may predict responsiveness to intervention. For instance, in non-constipated irritable bowel syndrome, a higher baseline abundance of *Collinsella aerofaciens* was identified as a potential biomarker for a positive clinical response to a specific probiotic strain (71). Translating this concept to obstetrics, future strategies must aim to identify similar predictive microbial or host-derived biomarkers in pregnant women. This would enable the stratification of individuals into likely responders and non-responders for targeted probiotic regimens or dietary prescriptions, moving beyond empirical supplementation toward precision nutrition and microbial therapy.

Personalization extends beyond microbial diagnostics to encompass dietary modulation as a foundational and modifiable lever. The diet-gut microbiota axis during pregnancy is a potent yet underutilized therapeutic target (15, 16). Specific dietary components, particularly fiber, are not only crucial for alleviating constipation but also shape a gut microbiota associated with favorable outcomes. Higher fiber intake is linked to gut microbial profiles that may support metabolic health. Evidence also suggests that increased dietary fiber and diversity are associated with a lower burden of antimicrobial resistance genes in the gut microbiome, a consideration of growing importance (48). Future interventions should integrate detailed dietary assessment to design personalized nutritional plans that selectively nourish beneficial autochthonous taxa, such as *Faecalibacterium* or *Lachnospira*, which are associated with positive metabolic influences in pregnancy (16). This dietary personalization, potentially guided by longitudinal microbiome profiling, could synergize with specific probiotic or synbiotic formulations to more effectively correct dysbiosis, relieve constipation, and create a healthier maternal metabolic and inflammatory milieu.

### 8.3 Future research directions

**Figure 3:**
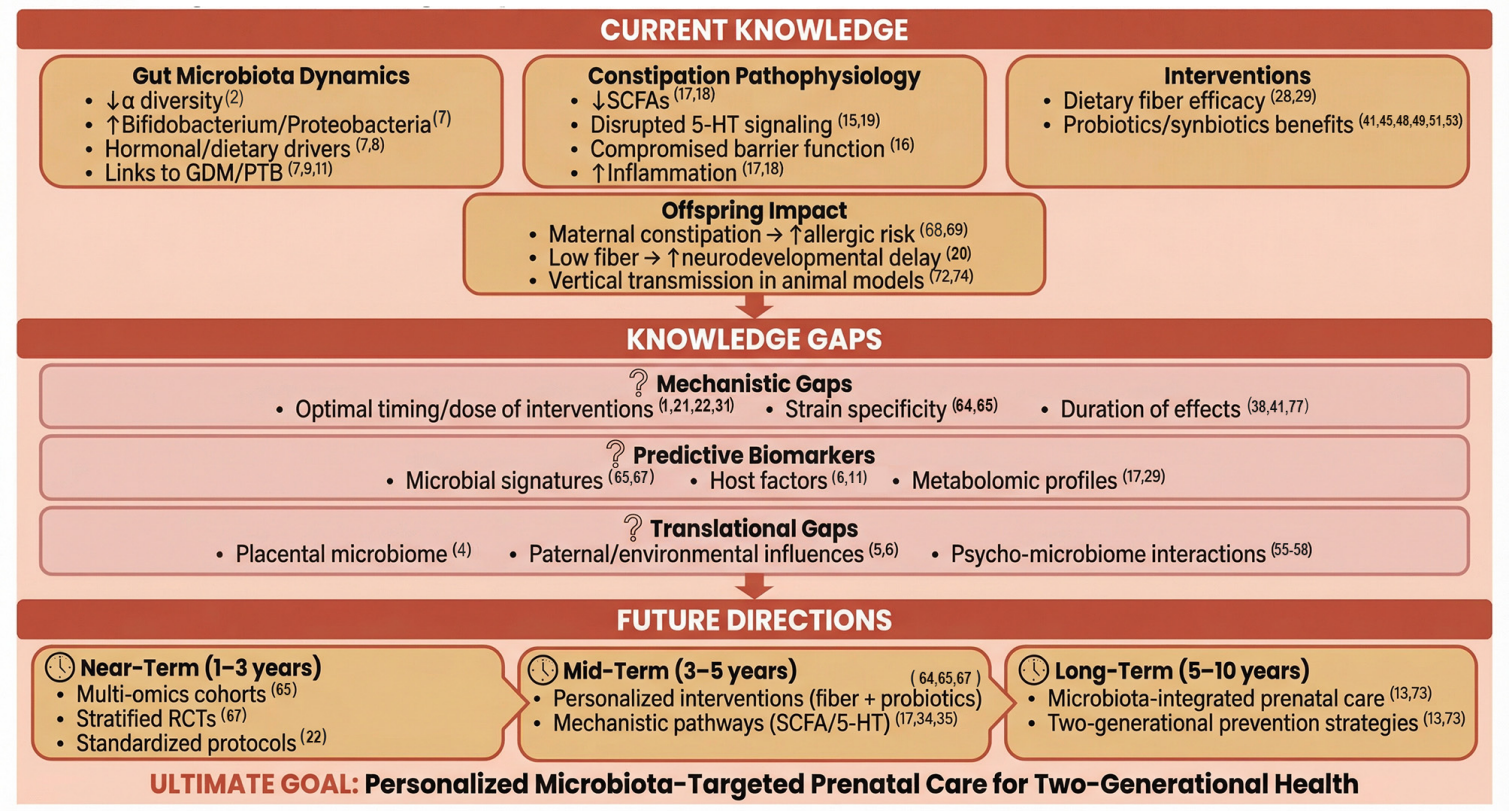
Knowledge gaps and future directions in gestational microbiome research. This conceptual framework summarizes the current state of knowledge, identifies unresolved questions, and proposes a roadmap for future research. Upper panel (current knowledge) synthesizes established evidence on gut microbiota dynamics during pregnancy, constipation pathophysiology, intervention efficacy, and offspring impacts. Middle panel (knowledge gaps) highlights three categories of unanswered questions: (i) mechanistic gaps regarding optimal intervention timing, type, dose, and duration; (ii) predictive biomarkers needed to identify responders vs. non-responders and predict complications; and (iii) translational gaps including placental microbiome, paternal influences, environmental factors, and psycho-microbiome interactions. Lower panel (future directions) proposes a timeline for research progress: near-term (1–3 years) focuses on large multi-omics cohorts and stratified RCTs; mid-term (3–5 years) develops personalized intervention packages and validates biomarkers; long-term (5–10 years) integrates microbiota assessment into routine prenatal care, transforming constipation management into a proactive, two-generational prevention strategy.

While the evidence summarized in this review establishes the maternal gut microbiota as a critical determinant of perinatal health, numerous questions remain unanswered. Figure 3 provides a conceptual roadmap organizing these knowledge gaps and proposing a timeline for future research. This framework guides the transition from current knowledge to personalized microbiota-targeted approaches in prenatal care.

Future research must prioritize large-scale, longitudinal cohort studies that deeply phenotype the maternal microbiome in conjunction with detailed dietary, clinical, and lifestyle data to identify robust predictive signatures for pregnancy complications and intervention response. Randomized controlled trials should be designed with stratification based on baseline microbial or metabolomic profiles to test personalized intervention packages, which may combine specific dietary fibers, prebiotics, and defined probiotic strains or consortia. Concurrently, mechanistic studies are needed to elucidate how microbial metabolites, such as those derived from fiber fermentation or bile acid metabolism, mediate improvements in intestinal motility and systemic inflammation during gestation. Ultimately, the goal is to develop clinical frameworks where assessment of the gut microbiota, alongside traditional clinical parameters, becomes integral to prenatal care. This will enable healthcare providers to offer evidence-based, personalized recommendations for diet and microbial supplementation, transforming the management of constipation from a symptomatic afterthought into a proactive strategy for optimizing the developmental origins of health and preventing disease across two generations. As visualized in Figure 3, this transformation requires a staged approach: near-term priorities focus on large multi-omics cohorts and stratified RCTs; mid-term goals involve validating predictive biomarkers and developing personalized intervention packages; long-term objectives integrate microbiota assessment into routine prenatal care, achieving the ultimate goal of two-generational health optimization.
